# Epithelial outgrowth through mesenchymal rings drives lung alveologenesis

**DOI:** 10.1172/jci.insight.187876

**Published:** 2025-01-07

**Authors:** Nicholas M. Negretti, Yeongseo Son, Philip Crooke, Erin J. Plosa, John T. Benjamin, Christopher S. Jetter, Claire Bunn, Nicholas Mignemi, John Marini, Alice N. Hackett, Meaghan Ransom, Shriya Garg, David Nichols, Susan H. Guttentag, Heather H. Pua, Timothy S. Blackwell, William Zacharias, David B. Frank, John A. Kozub, Anita Mahadevan-Jansen, Evan Krystofiak, Jonathan A. Kropski, Christopher V.E. Wright, Bryan Millis, Jennifer M.S. Sucre

**Affiliations:** 1 Department of Pediatrics,; 2 Department of Mathematics, and; 3 Department of Cell and Developmental Biology, Vanderbilt University, Nashville, Tennessee, USA.; 4 Department of Medicine, University of Minnesota, Minneapolis, Minnesota, USA.; 5 Department of Medicine, and; 6 Department of Pathology, Microbiology, and Immunology, Vanderbilt University Medical Center, Nashville, Tennessee, USA.; 7 Department of Veterans Affairs Medical Center, Nashville, Tennessee, USA.; 8 Department of Pediatrics, Cincinnati Children’s Hospital, Cincinnati, Ohio, USA.; 9 Department of Cardiology, Children’s Hospital of Philadelphia, Philadelphia, Pennsylvania, USA.; 10 Department of Bioengineering,; 11 Vanderbilt Biophotonics Center, and; 12 Department of Physics, Vanderbilt University, Nashville, Tennessee, USA.; 13 Department of Surgery, Neurological Surgery and Otolaryngology, and; 14 Biodevelopmental Origins of Lung Disease (BOLD) Center, Vanderbilt University Medical Center, Nashville, Tennessee, USA.; 15 Program in Developmental Biology, Vanderbilt University, Nashville, Tennessee, USA.

**Keywords:** Development, Pulmonology, Organogenesis

## Abstract

Determining how alveoli are formed and maintained is critical to understanding lung organogenesis and regeneration after injury. To study the cellular dynamics of this critical stage of lung development, we have used scanned oblique-plane illumination microscopy of living lung slices to observe alveologenesis in real time at high resolution over several days. Contrary to the prevailing notion that alveologenesis occurs by airspace subdivision via ingrowing septa, we found that alveoli form by ballooning epithelial outgrowth supported by contracting mesenchymal ring structures. Systematic analysis has produced a computational model of finely timed cellular structural changes that drive normal alveologenesis. With this model, we can now quantify how perturbing known regulatory intercellular signaling pathways and cell migration processes affects alveologenesis. In the future, this paradigm and platform can be leveraged for mechanistic studies and screening for therapies to promote lung regeneration.

## Abstract 2

With real-time, long-term 4-dimensional imaging of living lung slices, we have developed a new model of lung formation of epithelial ballooning through contractile mesenchymal rings.

## Graphical abstract

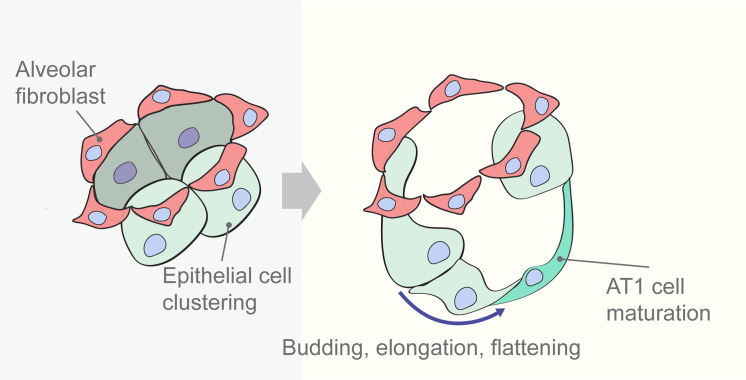

## Introduction

To generate the vast surface area required for gas exchange, lung organogenesis requires precisely coordinated intercellular signaling pathways to induce cell-type specialization and substantial changes in cellular structure. The gas-exchange epithelial surface expands more than 20-fold during the alveolar stage of development, spanning from 36 weeks’ gestation through early adolescence in humans, which is equivalent to postnatal days 5–28 (P5–P28) in mice. While the general timing of alveologenesis has been well defined histologically and transcriptomically, the precise cellular movements and dynamics of postnatal alveologenesis are not yet understood. Delineating these processes properly is critical to understanding how the refined architecture of the normal lung is built across the entire organ and the challenges involved in alveolar regeneration following lung injury.

While explants from relatively early prenatal stages of lung development (e.g., canalicular, saccular) are amenable to live imaging, the much-increased size and complexity of more mature postnatal tissue have, so far, made infeasible any extended, detailed live imaging during the main period of alveologenesis. With rare exceptions, previous findings on tissue architecture dynamics during the alveolar stage have been limited to inferences made from staged 2D (5–10 μm) histological sections, even after 3D reconstruction. There have been some live-imaging studies of postnatal precision-cut lung slices (PCLS), but photobleaching and phototoxicity kept the imaging periods short (<20 hours), and the use of epifluorescence widefield microscopy led to relatively shallow depth coverage (1–3). We have circumvented these technical hurdles using a tailored version of scanned oblique-plane illumination (SOPi) microscopy (4) for long-term (48–72 hours) high-resolution analysis of alveologenesis in PCLS. Using this 4-dimensional (4D) imaging platform on tissue from transgenic reporter mice, we tracked the stereotypical behaviors of epithelial and mesenchymal cells that drive the development of new alveoli. Abstracting data from this imaging system produced a computational model that quantifies the parameters of normal alveolar formation and expansion during early alveologenesis, for reference against disturbances caused by experimental manipulation or suffered under injury.

Here, we report how these 4D data from our new live-imaging system have led to the generation of a new model of lung development during the postnatal alveolar stage, based on epithelial cell migration and outgrowth through mesenchymal rings, in contrast with the current paradigm of subdivision by ingrowing alveolar septa. As proof of principle that this 4D system and computational analysis can quantitatively model both normal and perturbed tissue development, we applied small-molecule modulators of intercellular signaling pathways and processes critical for alveologenesis (as previously identified by studies with transgenic in vivo models and single-cell transcriptomics) (5–8). We report on the negative effects on alveologenesis caused by either broadly activating or inhibiting the Wnt signaling pathway, a known regulatory pathway of lung development (9). Second, because genetic and molecular interference with contractile processes occurring within the adjacent mesenchyme is known to disrupt alveolar lung development (8), we selectively inhibited myosin light chain kinase (MLCK), which revealed interactive effects between the cellular behaviors of the alveolar epithelium and mesenchyme, with inhibition of myofibroblast contraction and motility affecting epithelial cell movements and differentiation.

## Results

### PCLS provide an ex vivo model of alveologenesis.

**Figure 1:**
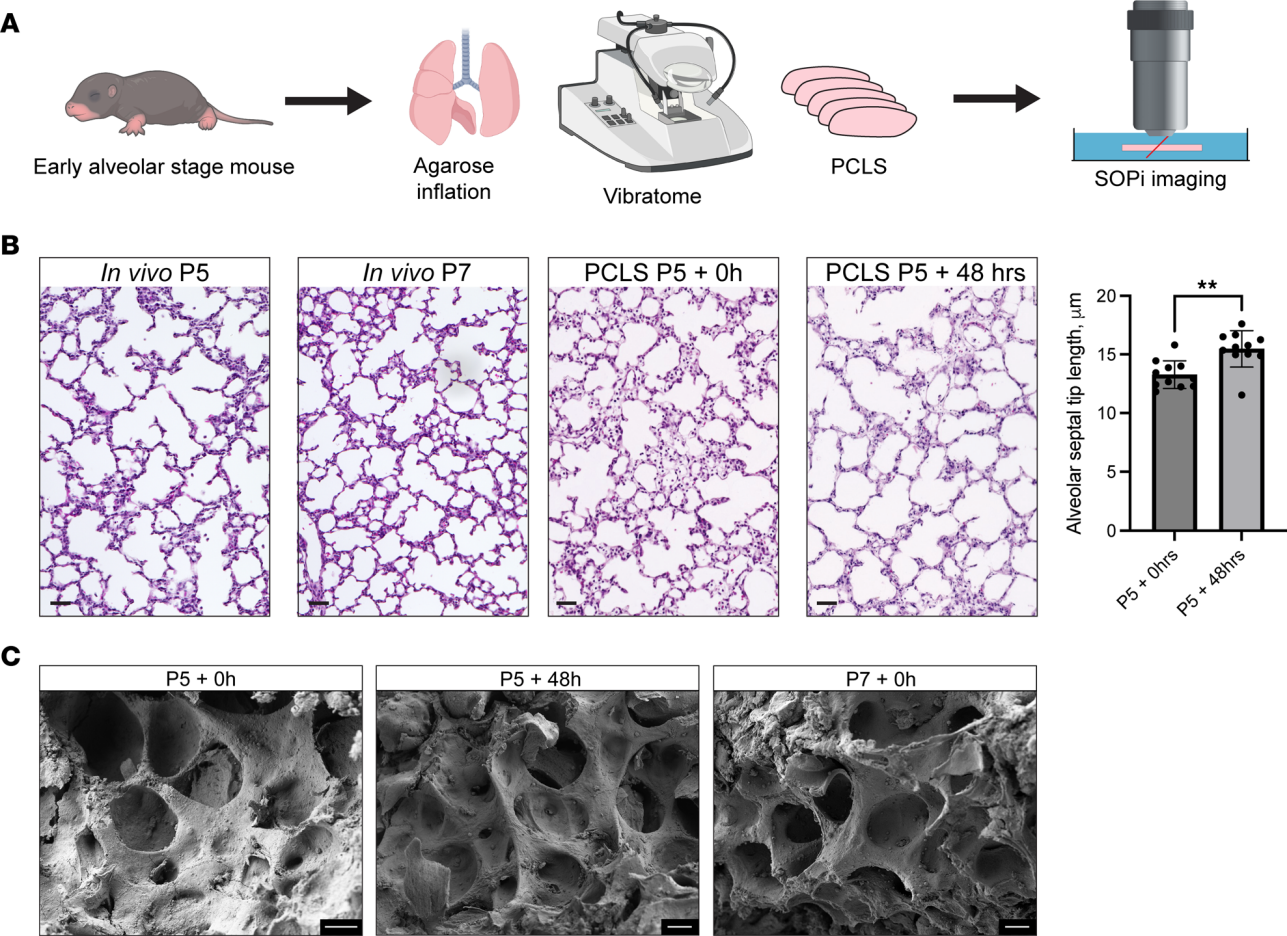
Precision-cut lung slices (PCLS) model of alveologenesis ex vivo. (**A**) Five-day-old mT/mG transgenic mice fluorescently reporting for alveolar epithelial, mesenchymal, or endothelial cells were imaged by scanned oblique-plane illumination (SOPi) microscopy. (**B**) PCLS were fixed immediately after preparation on P5, or after 48 hours in culture and stained with H&E. Alveolar septal tip length was calculated and compared between PCLS taken from the same lung, comparing P5 PCLS slices to P5 PCLS from the same lung after 48-hour culture (*n* = 6 mice from 2 separate litters, total of 11 PCLS per condition, with each point representing the average tip length values calculated from 9–10 images of an individual PCLS replicate), showing histological changes equivalent in many respects to H&E-stained sections from P5 or P7 mice, as reflected by alveolar septal tip length. Scale bars: 40 μm. **P *<* 0*.*001 by Student’s *t* test. (**C**) PCLS were imaged by scanning electron microscopy. Scale bars: 10 μm. Images are representative of SEM from *n* = 3 mice per condition/time point.

To directly visualize cellular changes associated with the development of new alveolar structures, we used ex vivo modeling of alveologenesis from PCLS derived from neonatal mice on P5, the transition point from the saccular to the alveolar stage (Figure 1A). As noted previously (10, 11), PCLS continue to develop and form new alveolar structures, with changes in tissue structure over 48 hours that mimic those seen over the same period of time in vivo (P5 to P7), as observed by 2D histological section analysis and scanning electron microscopy (SEM) (Figure 1, B and C, and Supplemental Figure 1; supplemental material available online with this article; https://doi.org/10.1172/jci.insight.187876DS1). We quantified changes in tissue complexity and structure in PCLS and in vivo in littermates by measuring alveolar septal tip length in H&E-stained sections (Figure 1B), observing equivalent expansions of distal airspace and surface area by SEM (Figure 1C), and morphometric quantification of airspace volume density (Supplemental Figure 1). While the production of PCLS (see Methods) leaves small amounts of gelled agarose in the large airways and some of the connected alveolar ducts, there was no FITC-labeled agarose detected in the distal parenchyma where we were imaging alveologenesis (Supplemental Figure 1). This finding circumvents concerns that there might be exogenous physical blockage of normal cellular morphogenesis processes with this system.

### Characteristic, rapid changes in epithelial cell shape and position generate an expansive surface area during alveologenesis.

**Figure 2:**
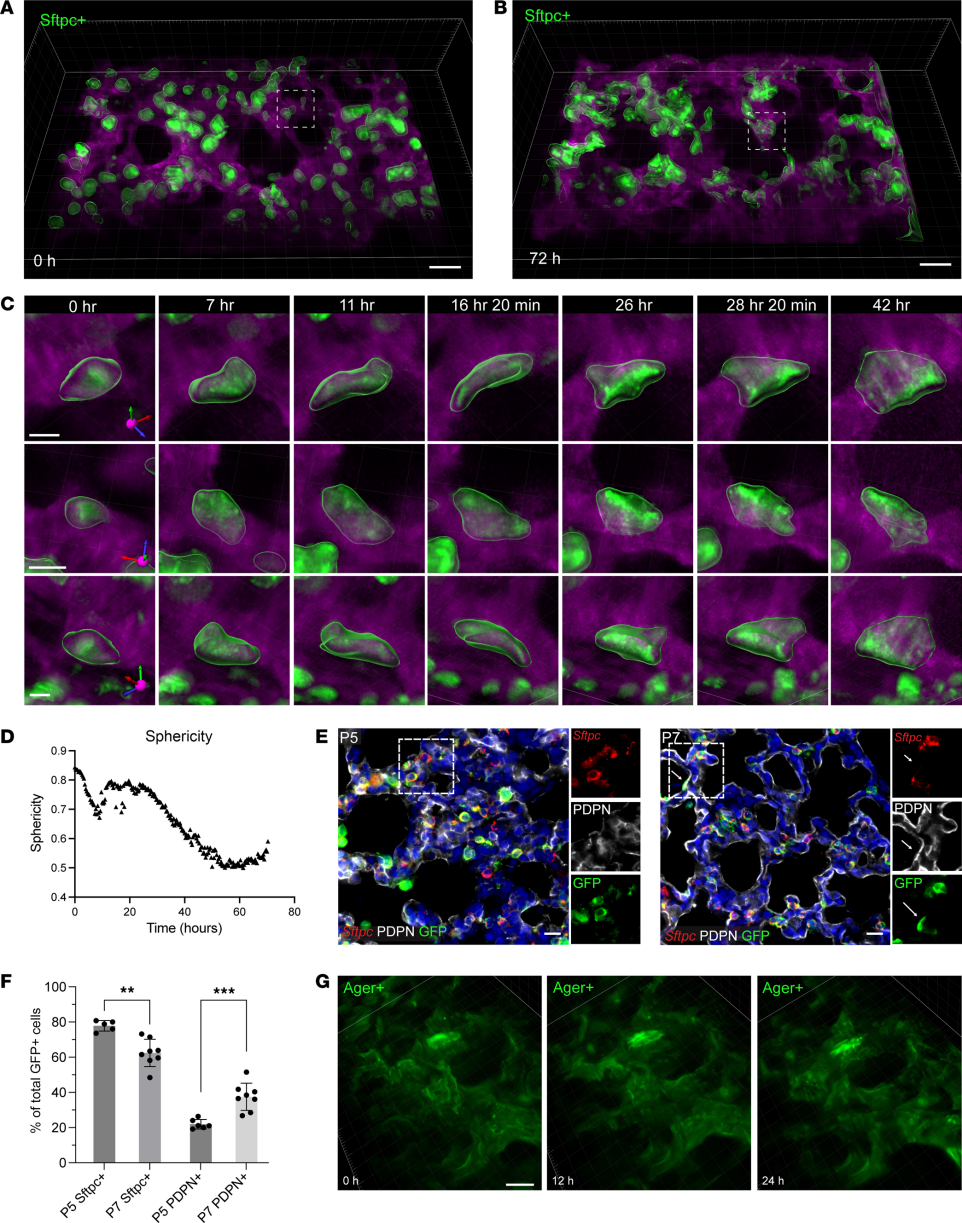
Alveolar type 2 cells undergo changes in cell shape that are associated with differentiation into alveolar type 1 cells. PCLS from mT/mG;Sftpc-CreER^T2^ mice (with tamoxifen given on P3 and P4) were volumetrically imaged and displayed as a 3D projection (GFP green, tdTomato magenta), representative of *n* = 9 imaging movies from 3 mice. (**A**) 3D projection of a still image from the start of the 72-hour imaging period. (**B**) 3D projection of a still image from the end of the same imaging period. Scale bars: 30 μm. (**C**) The same Sftpc-GFP–labeled cell viewed from 3 different orientations showing the dramatic cell-shape change from round to flat over 42 hours. Scale bars: 10 μm. (**D**) Quantification of sphericity of individual cells in **C** making this transition (representative of *n* = 83 cells counted). (**E**) Lungs from mT/mG;Sftpc-CreER^T2^ mice given tamoxifen on P3 and P4 and harvested on P5 (left) and P7 (right), immunostained with antibodies against GFP (green) and PDPN (white), analyzed by RNA in situ hybridization for *Sftpc* (red), with DAPI counterstaining (blue) to mark DNA. Scale bars: 10 μm. (**F**) Quantification of percentage Sftpc^+^ and PDPN^+^ cells among total GFP^+^ cells on P5 and P7, with each data point indicating the average value from 8–10 images of an individual mouse. **P < 0.01; ***P < 0.001 by 1-tailed Student’s *t* test (*n* = 5–6 mice on P5, 8 mice on P7). (**G**) Sequential still frames from 4D imaging of PCLS from mT/mG;Ager-CreER^T2^ mice (with tamoxifen given on P3 and P4). Scale bars: 30 μm. Representative of *n* = 9 imaging movies from 3 mice.

The differentiation of alveolar epithelial cells from alveolar type 2 epithelial (AT2) cells into alveolar type 1 (AT1) cells involves large-scale cell-shape alteration — from cells that are more rounded to extremely thin and outspread — with this change in shape allowing these gracile cells to assume gas-exchange function (12). To follow the dynamics of specific cells during alveologenesis, we used mT/mG Cre-switched transgenic mice that express membrane-bound tdTomato (mT) before Cre excision but membrane-bound green fluorescent protein (mG) afterwards (13). Mice with an inducible reporter for AT2 cells (Sftpc-CreER^T2^) (14) received intraperitoneal tamoxifen on P3 and P4. We followed the changes in cell shape at single-cell resolution by tracking mG fluorescence in Sftpc^+^ cells in PLCS made on P5, imaged over a depth of 100–150 μm through the tissue, which allowed tracking and shape quantification of individual cells from multiple angles (Figure 2, A–C). We measured sphericity, a metric of relative roundness/flatness to quantify changes in shape of individual cells (Figure 2D). While some GFP^+^ cells remained round and relatively stationary, many cells underwent substantial outward ballooning and flattening during alveolar-structure formation. Over 72 hours, approximately one-quarter of Sftpc-traced cells at P5 undertook a complete, fairly rapid round-to-flat transition as measured by live imaging (Supplemental Figure 2 and Supplemental Video 1). Analysis of P14 PCLS during later alveologenesis showed similar changes in cell shape, albeit in decreased percentages and frequency, with 24% of Sftpc^+^ cells flattening, and a significant decrease in number of alveologenesis events per hour when compared with PCLS from P5 mice (Supplemental Figure 2). From the beginning of any imaging period, there were variable lag periods among the cells, with flattening/extending epithelial movements occurring essentially asynchronously across the tissue. Once any cell began to spread out, however, the flattening period was consistently estimated at approximately 42 hours (Figure 2D).

In this Sftpc lineage trace, a transition from a more spherical to a flatter cell shape was associated with loss of *Sftpc* expression and gain of podoplanin (PDPN), a hallmark of AT1 cells, within each flattened cell (Figure 2, E and F). With tamoxifen induction on P3 and P4, 78% of GFP^+^ cells coexpressed *Sftpc* on P5, with 22% of GFP^+^ cells negative for *Sftpc* and already flattened by P5 and expressing PDPN (Figure 2, E and F, and Supplemental Figure 2). Both fixed PCLS from P5 mice after live imaging for 48 hours and also lungs from P7 mice that received tamoxifen induction on P3–P4 had 39.7% of all GFP^+^ cells that were PDPN^+^ and *Sftpc*^–^, providing strong supporting evidence for associating AT2-to-AT1 differentiation with shape change at this stage. The 60.1% of GFP^+^ cells that were SFTPC^+^PDPN^–^ at P7 (and after 48 hours of imaging) retained AT2 shape and expression characteristics. A rare (<1%) population of cells was positive for both markers (possibly representing an intermediate cell state), and none of the GFP^+^ cells were negative for both SFTPC and PDPN. While a small number of GFP^+^ cells were PDPN^+^ and appeared flattened at the start of imaging, none of these flattened cells became rounder over time. With imaging PCLS from Ager-CreER^T2^;mT/mG to label AT1 cells, nearly all the GFP^+^ cells were relatively flat at that start of imaging at P5 (after tamoxifen on P3 and P4), with very little movement of the Ager^+^GFP^+^ cells over 48 hours. None of these Ager^+^GFP^+^ cells changed shape to become round over the multiple-day imaging period (Figure 2G).

**Figure 3:**
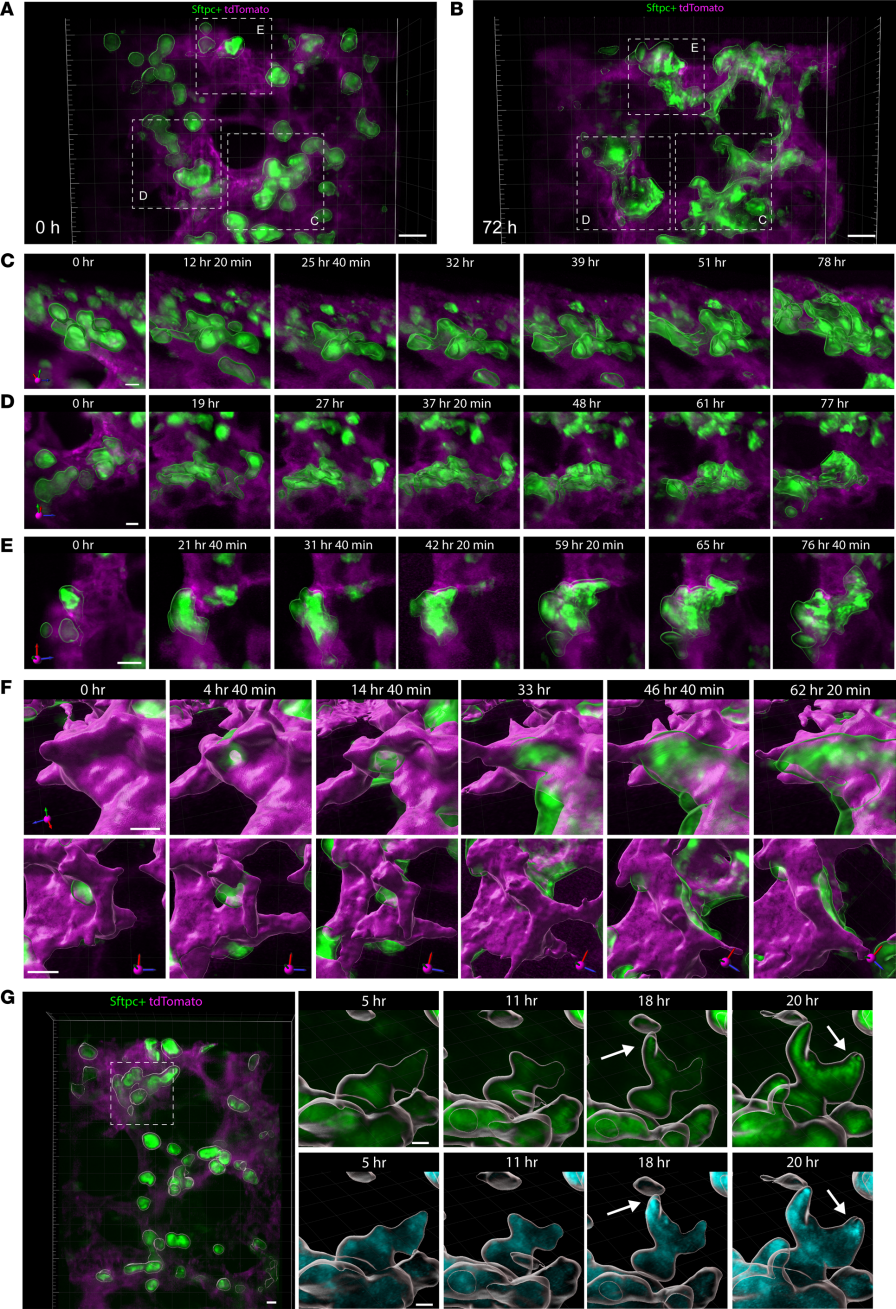
Alveologenesis is characterized by epithelial ballooning outgrowth. (**A**) PCLS from mT/mG;Sftpc-CreER^T2^ mice (with tamoxifen given on P3 and P4) were volumetrically imaged and displayed as a 3D projection (GFP green, tdTomato magenta). Left: 3D projection of a still image from the start of the 72-hour imaging period. Scale bars: 20 μm. (**B**) 3D projection of a still image from the end of the same imaging period. (**C**–**E**) Insets of 3 different areas of epithelial cells clustering with ballooning outgrowth and elongation, as representative areas of this process, which occurs asynchronously across the PCLS. Scale bars: 10 μm. (**F**) Imaris surface rendering of Sftpc-GFP^+^ epithelial cells (green) moving through ring-like structures (magenta) and expanding in the formation of neo-alveoli shown in 2 perspectives. Scale bars: 10 μm. (**G**) PCLS from mT/mG;Sftpc-CreER^T2^ mice given tamoxifen on P3 and P4 and harvested on P5, live imaged with a probe for F-actin (blue, segmented to only show GFP^+^ cells), demonstrating polymerized actin (white arrows) at the leading edge of the epithelial cell just before movement. Scale bars: 10 μm. All images are representative of *n* = 9 movies from 3 mice, each with 3 regions of interest imaged per experiment.

In addition to individual cell flattening, live imaging revealed complex cellular movements of individual and groups of cells, with multiple asynchronous episodes of GFP^+^ cells clustering together, ballooning, and flattening in the formation of a neo-alveolus (Figure 3, A–F, and Supplemental Video 1). We observed similar epithelial cellular movements using multiple Cre drivers of mG expression in epithelial cells, such as *Shh* and *Nkx2.1* (Supplemental Figure 3, A and B). There are many potential mechanisms that coordinate shifts in cell shape and movement, with convergence on F-actin polymerization as a primary driver of cell motility. Using a probe that labels F-actin within living cells, we found polymerizing actin fibers at the leading edge of extending AT2 cells just prior to their perceptible movement, and absence of polarized actin polymerization in cells that were not migrating or changing shape (Figure 3G). Notably, epithelial clustering, outward extrusion, and flattening occurred with relative lack of influence from cell proliferation, based on the measurement of cell division using live-nucleus tracking or immunostaining for Ki67, with the latter showing approximately 1% of Sftpc^+^ cells that were Ki67^+^ (Supplemental Figure 3, C and D). These data are consistent with only a minor proportion of proliferating AT1 or AT2 cells being detected at this stage by analysis of a prior published transcriptomic atlas of the developing lung (5) (Supplemental Figure 3E).

### Forming alveolar structures requires alveolar mesenchymal cells arranged in extensively connected and dynamic ring-like structures.

**Figure 4:**
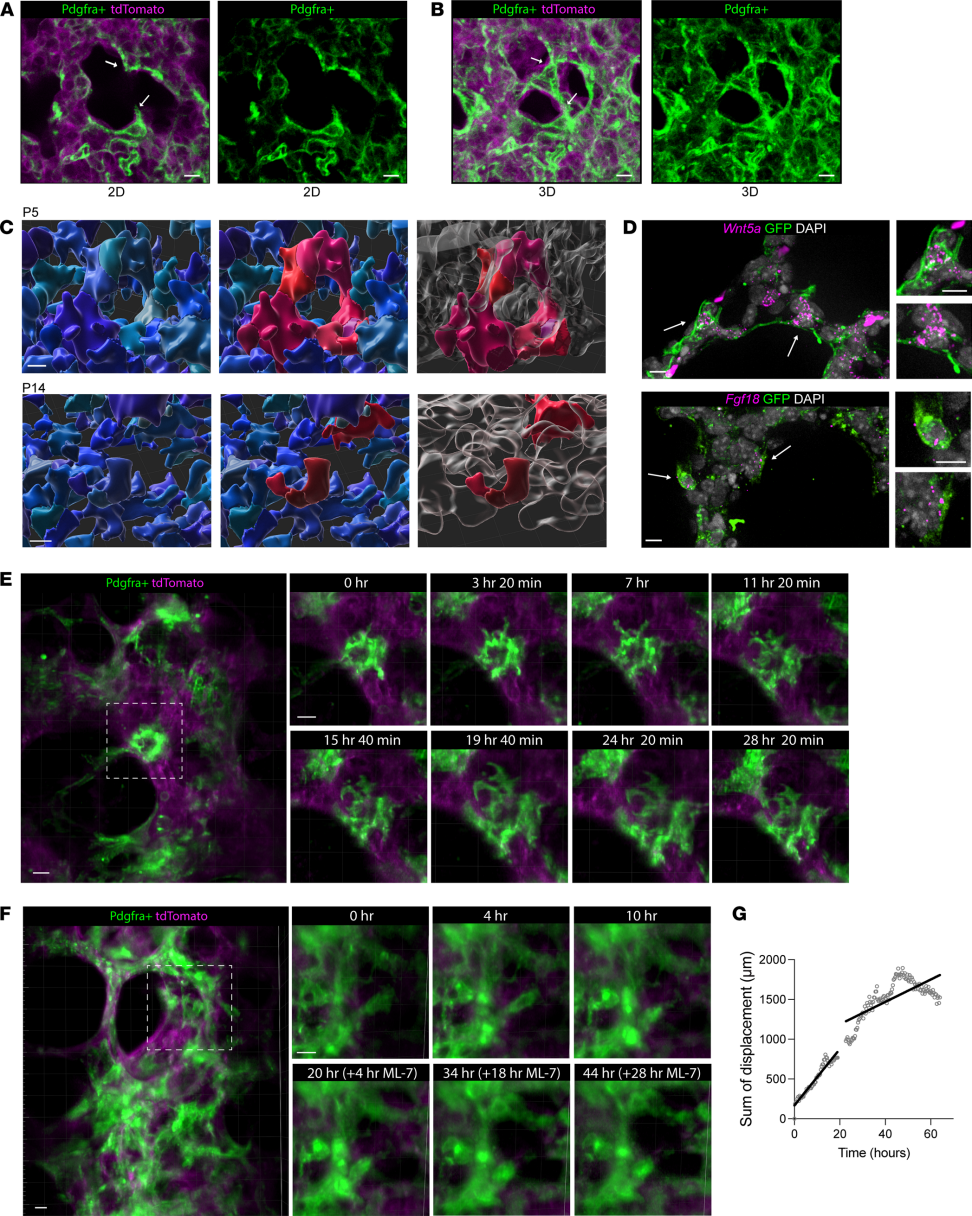
Mesenchymal ring structure dynamics and contraction are required for alveologenesis. PCLS from mT/mG;Pdgfra-Cre mice were volumetrically imaged via SOPi for 72 hours. Representative of *n* = 5 mice, each with 3 regions of interest imaged per experiment Comparison of (**A**) a single plane with (**B**) a volumetric rendering of the *Z*-stack from the same area demonstrates that putative “septal tips” (white arrows) are in fact part of a 3D ring structure. (**C**) Imaris-based surface rendering of thick, tissue-cleared sections from the lungs of mT/mG;Pdgfra-Cre mice on P5 and P14 demonstrates gradual loss of the ring structure over the course of alveologenesis, with Pdgfra^+^ cells in blue (left) showing the complex ring network, with individual rings highlighted in red (middle) and shown in the context of adjacent growing airspaces (right). (**D**) Whole-mount tissue-cleared RNA in situ hybridization of thick sections with probes for hallmark genes of alveolar myofibroblasts *Wnt5a* (top) and *Fgf18* (bottom) demonstrate colocalization with GFP (green). (**E**) Still frames from excerpt of larger 72-hour 4D imaging of lungs from mT/mG;Pdgfra-Cre mice demonstrate the dynamic movement of individual cells to form ring structures and gradual contraction of individual rings over time. (**F**) PCLS from these mice administered the ML-7 inhibitor after 20 hours of imaging dramatically decreased the displacement of Pdgfra^+^ cells (inset) quantified by a change in rate of movement of individual cells over time (**G**). All scale bars: 10 μm.

We used mT/mG;Pdgfra-Cre mice to label alveolar myofibroblasts, as done previously (15, 16). In 3D, P5 PCLS from these tissues demonstrated an extensive network of connected ring-like structures, each formed by 3 to 5 Pdgfra-traced cells (Figure 4, A–C), with coexpression of previously identified hallmark genes of these specialized alveolar mesenchymal cells, including *Fgf18* and *Wnt5a* (Figure 4D). In analyzing single optical planes within the context of several individual *Z*-stacks, any Pdgfra^+^-traced alveolar fibroblasts that appeared in 2D as putative “tips of in-growing septa” were revealed in fact not to be extending into “free space,” but rather were part of the mesenchymal ring-like structures (Figure 4, A and B, and Supplemental Video 3). In both fixed 3D sections and live imaging at P5, P7, P10, and P14, ring shape and diameter evolved during the alveolar stage (Figure 4C and Supplemental Video 4). Live imaging showed 2 distinct kinds of physical movements of these cells: migration of Pdgfra^+^ cells around the ring scaffold to form new ring-like structures (Supplemental Video 3) and a subtle, yet consistent, relative contraction of ring diameter over the 72-hour imaging period (Figure 4E). Many Pdgfra-traced ring structures were present when imaging started, and some additional rings formed during imaging (Figure 4E and Supplemental Video 3). We characterized the structure of these rings at multiple time points across alveologenesis in vivo and ex vivo: P5, P7, P10, and P14. Over time, the number of rings per unit volume decreased, with the Pdgfra^+^ cells losing their ring-like integrity by P14 (Figure 4C, Supplemental Figure 4A, and Supplemental Video 4). Across multiple time points, the ring architecture differed at the start of live imaging between P5, P10, and P14 lungs, and these differences were observed in vivo in P5, P10, and P14 lungs (Supplemental Figure 4, B and C). Surprisingly, the ring-shape changes ex vivo for 48–96 hours did not mimic the gradual loss of ring shape in vivo when comparing slices imaged over the same time period. Possibly, modification of the cells comprising the rings requires circulating macrophages absent from this ex vivo system (17), or that other mechanical forces that are not present (see Discussion). The subtle decrease in diameter observed in the ring-like structures suggested that myofibroblast contraction was involved. To test this, we performed live imaging in the presence of the ML-7 inhibitor of MLCK, which caused a complete arrest of both ring contraction and GFP^+^ cell movement in general (Figure 4, F and G). With Sftpc lineage–traced PCLS, ML-7 decreased AT2-to-AT1 differentiation; there were almost no GFP^+^PDPN^+^ cells in PCLS treated with ML-7 (Supplemental Figure 4D). As epithelial cells do not express MLCK, we interpret this result to suggest that the migratory movement of Pdgfra^+^ cells and the contraction of the myofibroblast rings are necessary to support the epithelial ballooning movements (Supplemental Figure 4, D and E).

Under control conditions, epithelial cells always clustered adjacent to the ring-like structures, followed by directional extrusion through the ring, extension, and flattening, collectively undergoing an outward ballooning movement to create a new alveolar lumen (Figure 3F and Supplemental Video 5). With extrusion, the soma of each epithelial cell became displaced away from the ring by 60–100 μm. This distance approximates the normal alveolar diameter in P5 mice (18), suggesting that the ballooning process is subject to “self-organizing constraints” that establish a uniformly appropriate initial alveolar size and structure. Without any apparent difference across the 6.16 million μm^3^ (one ROI of 350 μm × 220 μm × 80 μm; 2–3 ROIs imaged in each PCLS) tissue volume imaged from multiple PCLS, there was a reproducible sequence of epithelial cell clustering and extrusion, followed by elongation and flattening (Figure 3, C–E, Supplemental Figure 3A, and Supplemental Video 1), although as already mentioned, alveologenesis events were broadly asynchronous across any single PCLS (Supplemental Video 2). Notably, alveoli formed in single-extrusion and adjacent dual-extrusion structures, as well as more rarely in conjoined groups of 3 to 5 alveologenesis events (Figure 3, C–F, and Supplemental Videos 1 and 2). The latter is consistent with alveologenesis often involving temporally concurrent formation of multiple adjoined alveoli with common separating walls (19).

### Endothelial cells assemble a complex vascular network during alveologenesis ex vivo.

**Figure 5:**
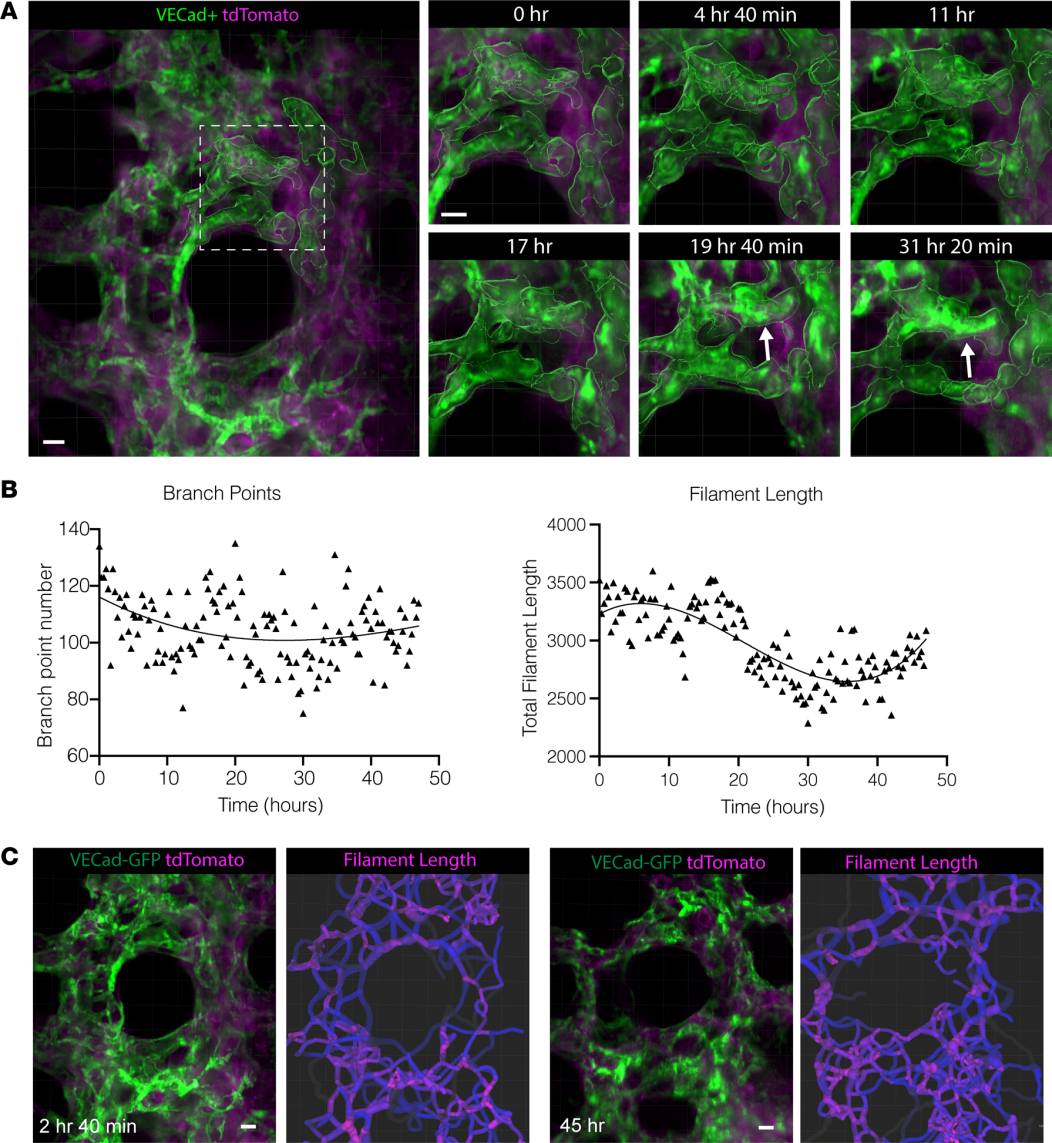
Endothelial cells form a dynamic complex vascular network during alveologenesis. PCLS from mT/mG;Vecad-Cre mice were volumetrically imaged by SOPi for 48 hours. All images are representative of *n* = 9 movies from 3 mice. (**A**) Still frames from this imaging period (inset) show movement and elongation of some individual cells (white arrows) and changes in network complexity as quantified by (**B**) number of branch points and length of individual filaments. (**C**) Filament length at 2 different points in time during the imaging period represented graphically with a colorimetric scale (shorter filaments in pink, longer filaments in blue). All scale bars: 10 μm.

The formation of an alveolus as a functional gas-exchange unit requires the apposition of epithelial and endothelial cells, and with AT2-to-AT1 differentiation occurring in parallel with the differentiation of general pulmonary capillaries (gCap/CAP1 cells) toward specialized alveolar capillaries (aCap/CAP2 cells) (6, 20, 21). Using mT/mG;Vecad-Cre to label endothelial cells allowed definition of changes in the shape and movements of individual cells and the capillary network overall. Some of the endothelial cells migrated and flattened in a pattern similar to that observed previously in differentiating epithelial cells (Figure 5A). While not being able to form a functional circulatory network ex vivo, the GFP^+^ endothelial cells did demonstrate an intricate network, with maintained vascular connectivity even while the cells rearranged. Quantifying the growing complexity of this capillary network demonstrated marked changes in the length of individual segments, without notable changes in the number of connections between segments (Figure 5, B and C), consistent with prior work in rats quantifying the changes in the capillary network during the alveolar phase (22).

### Modulating Wnt signaling greatly impairs epithelial migration and outgrowth in alveologenesis.

**Figure 6:**
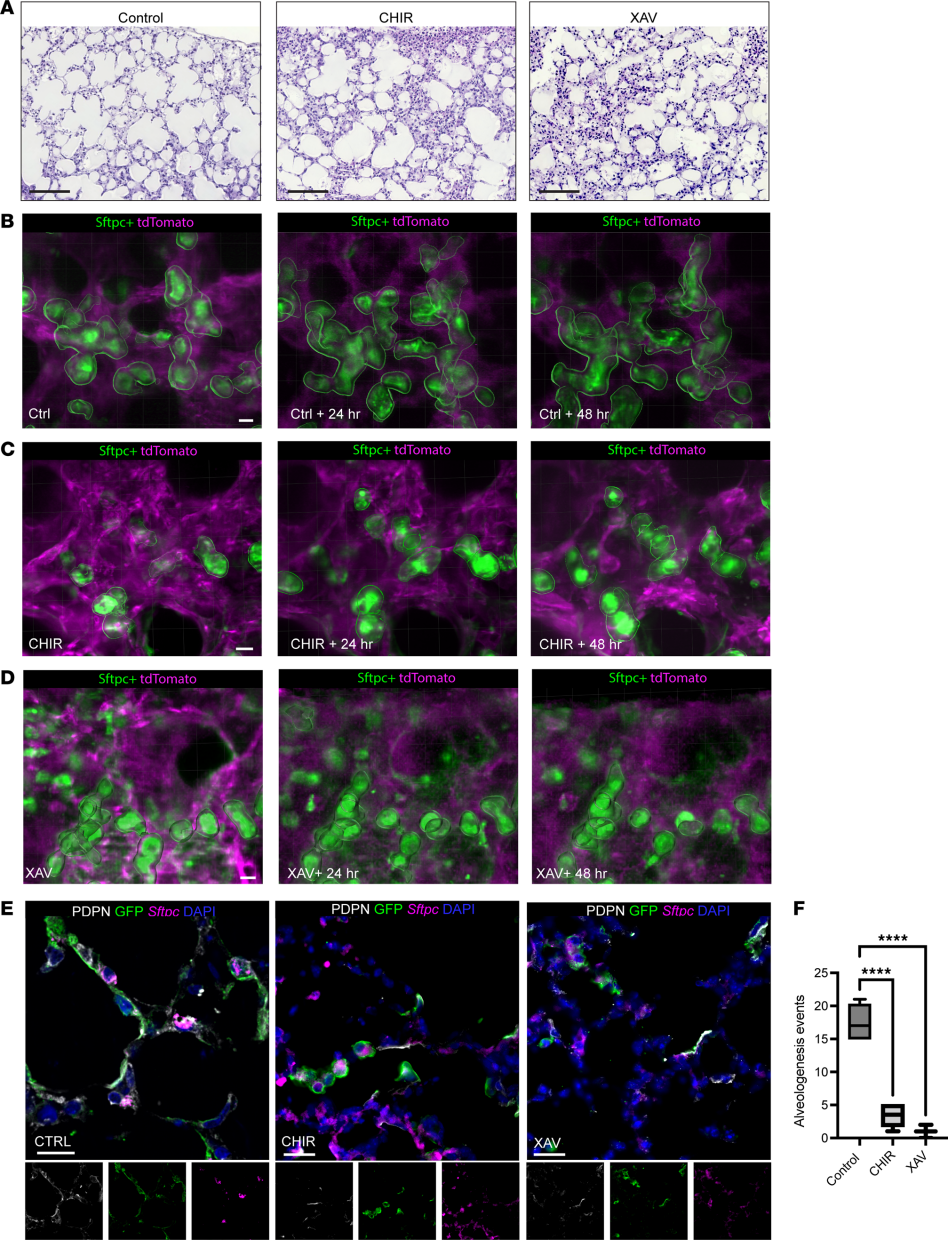
Modulation of the Wnt pathway disrupts alveologenesis, with decreased epithelial ballooning movements and changes in shape. (**A**) H&E staining of PCLS made on P5 and cultured for 72 hours under control conditions and with the addition of Wnt activator CHIR-99021 or Wnt inhibitor XAV-939 demonstrate abnormal alveologenesis with both modulators. Scale bars: 100 μm. Still projections from (**B**) control, (**C**) CHIR-99021–, or (**D**) XAV-939–treated PCLS imaged over time. Scale bars: 10 μm. (**E**) Control, CHIR-, or XAV-treated PCLS from mT/mG;Sftpc-CreER^T2^ mice were immunostained for GFP (green) and PDPN (white) and analyzed by RNA in situ hybridization for *Sftpc* (magenta), with DAPI counterstaining (blue) to mark DNA. Scale bars: 10 μm. (**F**) Individual alveologenesis events by epithelial cells were scored in a blinded manner from PCLS from CHIR (Wnt activator), XAV (Wnt inhibitor), or vehicle control. ****P *<* 0.001 by 1-way ANOVA followed by Dunnett’s multiple-comparison test with Bonferroni’s correction. *n* = 5 for CHIR exposure. *n* = 7–9 movies from 3 mice per condition, with a minimum of 5 PCLS immunostained per group.

A key feature of the SOPi system is that it provides access for applying small-molecule pathway modulators onto the living tissue during imaging. To determine whether this platform could efficaciously reveal normal and abnormal developmental processes, we imaged PCLS from Sftpc-CreER^T2^;mT/mG mice treated with modulators of Wnt signaling, a pathway already well connected to alveologenesis (9, 23). PCLS were cultured with CHIR-99021 (hereafter CHIR; to activate canonical Wnt signaling) or XAV-939 (a pan-Wnt signaling inhibitor through inhibiting tankyrase). With both perturbations, PCLS had significantly fewer alveologenesis events than controls (Figure 6, A–D), with Wnt activation or inhibition confirmed by immunofluorescence for nuclear, activated β-catenin (Supplemental Figure 5A). With Wnt activation via CHIR, some epithelial cells began to elongate and begin soma movement but then reverted to their original shape and position (Figure 6, B and C, and Supplemental Video 6). Using nuclear tracking as a proxy for cellular displacement, CHIR application caused epithelial cells to move faster than controls (Supplemental Figure 5B) but not with consistent, vectored movement as quantified by decreased directional processivity. Indeed, imaging data revealed numerous cells engaged in oscillatory back-and-forth behavior while failing to complete extrusion and ballooning movements (Supplemental Video 6). By contrast, global Wnt inhibition caused epithelial cells to exhibit little to no movement (Figure 6C and Supplemental Video 6). In this system, perturbation of an intercellular signaling pathway essential to normal lung development resulted in demonstrable and quantifiable impairment in alveolar epithelial cell movement (Figure 6F) and decreased differentiation, as demonstrated by RNA ISH for *Sftpc*, and immunofluorescence for PDPN and GFP, with almost no GFP^+^PDPN^+^ cells (<1% of all GFP^+^ cells) with either Wnt activation or inhibition (Figure 6E). Globally activating or inhibiting Wnt signaling resulted in loss of the tissue structural changes in normal alveologenesis, providing some insight into how dysregulated Wnt signaling with injury might impair lung development and regeneration.

### Computational modeling derived from 4D imaging for rigorous parameterization of alveologenesis.

**Figure 7:**
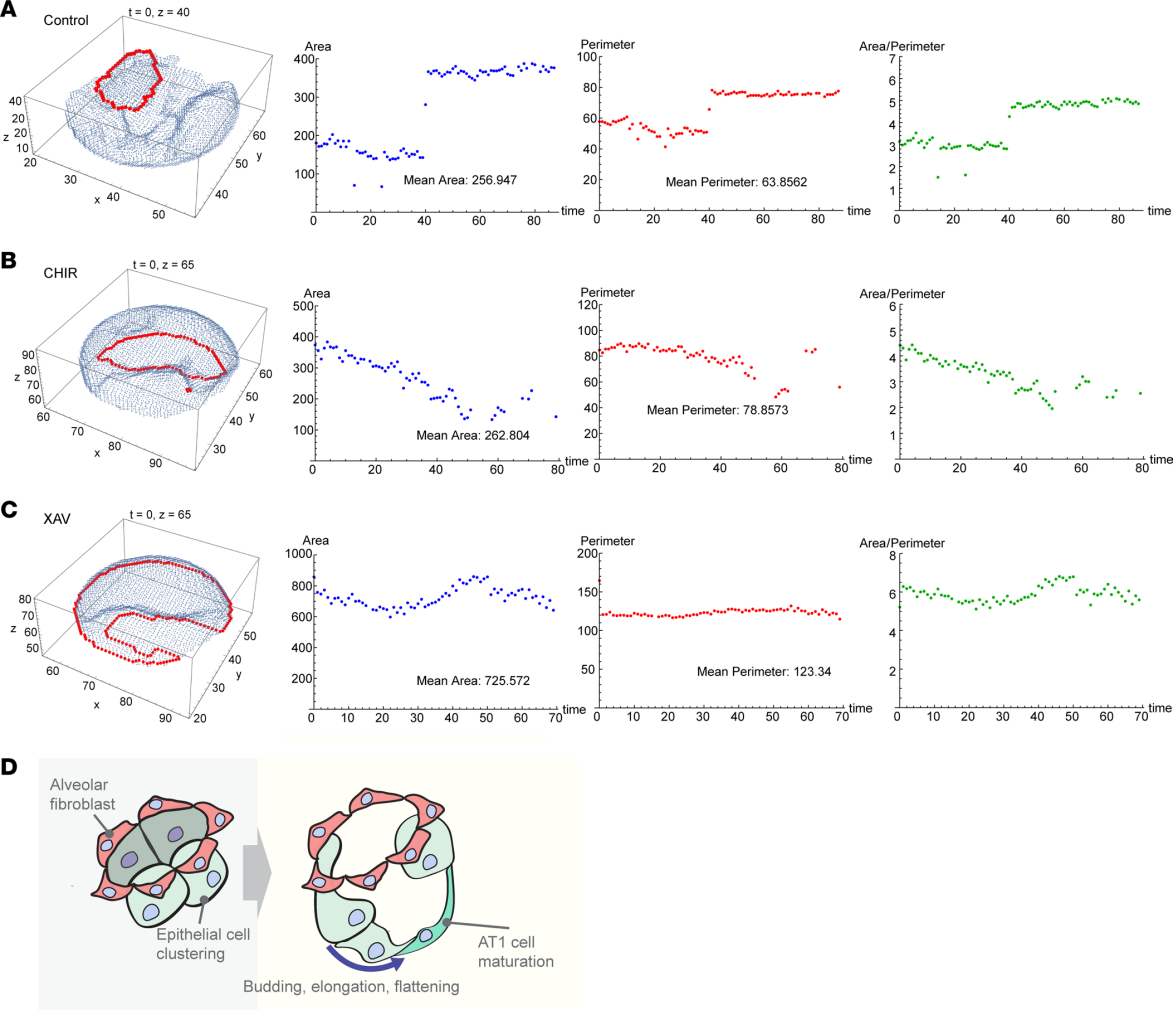
Computational modeling of alveologenesis based on membrane/cell shape–tracking data parameterizes epithelial outgrowth. (**A**–**C**) Computational modeling of representative single alveolar buds from (**A**) control, (**B**) CHIR-99021–treated (Wnt activator), and (**C**) XAV-939–treated (Wnt inhibitor) PCLS imaged over time. Area, perimeter, and the area/perimeter ratio (a proxy of complexity) were calculated. Data shown are representative of individual alveoli from *n* = 4 movies that were analyzed per condition. (**D**) Proposed schematic of alveologenesis characterized by epithelial outgrowth from a foundational mesenchymal ring.

To quantify and parameterize normal alveolar growth, we constructed a computational model from our membrane fluorescence and cell shape–tracking data. Abstracting 3D fluorescence data from multiple alveoli into a computational matrix (Figure 7, A–C) allowed accurate modeling of the expanding epithelial perimeter and alveolar area over time (Figure 7, A–C). Building and testing this model over numerous alveologenesis events under control conditions fits well with the observed structural changes of epithelial outgrowth and alveolar expansion (Figure 7D). PCLS treated with CHIR or XAV-939 showed very little perimeter expansion compared to controls. Overall, these data are consistent with the observations above that either up- or downregulating Wnt signaling greatly disturbs epithelial extrusion, differentiation, and alveolar growth.

## Discussion

Taken together, our 4D imaging data of postnatal lung development strongly support a fundamental shift in our understanding of alveologenesis as being driven by epithelial cell collection at interconnected mesenchymal ring–support structures, with subsequent directional epithelial extrusion through the rings, which is tightly accompanied by the extensive spreading and flattening that mark differentiation into mature AT1 cells. These observations are consistent with the fishnet-like mesenchymal web recently proposed to support alveolar development (15) and with mesenchymal ring contraction being essential for alveologenesis (8). Our findings suggest an alveolar-stage process that resembles the budding movements previously reported for the earlier saccular stage (E15.5–E18.5), wherein epithelial cell protrusion drives differentiation in explants (24). We also note concordance of our data with a previous report of live imaging of PCLS at P3, in which antibody-labeled epithelial cells were observed to cluster and extend at the end of the saccular stage (1). While the scale of epithelial surface area expansion during the alveolar stage is 20-fold greater than the saccular stage, requiring that the cellular dynamics accommodate much more than just a continuation of a simpler earlier budding process, it seems possible that the observed cellular movements and changes in epithelial cell shape in all of these studies do represent a continuum of cellular behavior across developmental time, culminating in the highly dynamic alveolar stage. Indeed, competent alveologenesis results in multiple alveoli growing to have shared walls, with a closely apposed vascular network for gas exchange. Also noteworthy are the rapid movements of *Pdgfra*^+^ cells between ring structures in the formation of new rings, and the requirement of ring contraction for epithelial outgrowth, which could only be appreciated in long-term volumetric imaging on thick PCLS sections.

Despite searching extensively across more than 2,000 hours of live-imaging data, we found no instances representing dynamically subdividing epithelial-mesenchymal septal walls or evidence that airspace subdivision during alveologenesis occurs by cellular ingrowth, and we currently conclude that such processes are rare or nonexistent, and therefore are at least a very minor contributor. Rather, our model for the predominant mechanism is that ring-proximal AT2 epithelial cell clustering is followed by directional cellular extrusion, ballooning, and flattening, with the flattening changes coinciding with the observed transition from AT2 to mature AT1 cells. This arguably radical departure from septal-ingrowth models of alveologenesis should afford a substantial and appropriate refocusing of the types of biological questions to be pursued. As described in the Results, comparing simultaneously acquired single-plane and *Z*-stack volumes renders a plausible explanation for how the septal invagination model may have misleadingly arisen as an artifact associated with single-plane live imaging, 2D histologic sections, and a limited capacity for extensive 3D reconstruction from wide-field microscopy. With this mesenchymal ring–epithelial outgrowth model, we propose future investigation of principal issues such as the mechanisms by which stabilization but then disappearance of the mesenchymal ring (17) acts to transiently scaffold the final parenchymal structure, and whether or not the 60- to 100-μm displacement distance of flattening and ballooning epithelial cells sets the initial neo-alveolar diameter. Additional critical topics include describing how differentiating epithelial cells come to align with the forming capillary network, and how the interplay of various intercellular signaling pathways drives the requisite changes in epithelial cell location and shape. Such questions and others connected to them represent the next frontier in our understanding of lung development as well as the challenges needing to be overcome to engage fully efficacious regeneration after injury.

Mesenchymal cells are known important sources of Wnt ligands during early alveologenesis, and there is particularly precise spatiotemporal patterning of *Wnt2* and *Wnt5a* expression, with Wnt5a being produced specifically in the alveolar myofibroblasts, along with other Wnt ligands (5). We speculate that relatively steep local gradients of Wnt promote AT2 cell aggregation and initial extrusion near the mesenchymal ring. Subsequent cell movement away from the ring results in decreased Wnt exposure, allowing proper AT2-to-AT1 differentiation and flattening. We found that Wnt inhibition prevented alveolar epithelial cell migration and extrusion at the earliest stages of outgrowth ballooning. As global Wnt activation also impaired alveologenesis, it is plausible that CHIR overwhelmed the spatial anisotropy of the mesenchymal ring–sourced Wnt gradient, preventing cellular movement away from “high-Wnt” regions. In this model, cell displacement away from ring-sourced Wnt to lower-Wnt regions would allow a spatiotemporally appropriate rise to dominance of other signaling pathways that orchestrate rapidly progressive AT1 differentiation, such as BMP signaling (25). Such speculation is consistent with the knowledge that waves of Wnt signaling are required for alveologenesis and that Wnt withdrawal is required for AT2-to-AT1 differentiation in vitro (9). Of note, in this system, we only observed AT2-to-AT1 differentiation by lineage tracing with Sftpc-Cre. While the possibility of AT1 cells “back differentiating” into AT2 cells has been suggested by recent reports labeling cells expressing AT1 hallmark genes at early prenatal time points (26), our lineage-tracing analysis using Ager-Cre (with tamoxifen given on P3 and P4 to label mature AT1 cells) revealed no instances of flattened GFP^+^ cells becoming round. Additionally, recent work supports the idea that some AT1 hallmark genes, e.g., *Hopx*, may be relatively less specific in early life (27). Future work with this live-imaging system envisions the capacity to label multiple cell types within the same mouse to further characterize the transitions in cell state and shape, and the precise relationships between multiple cell types in organizing alveolar structures.

No model is without limitations — indeed, these results rely heavily on observations of lung slices ex vivo and submerged in liquid, and therefore without an air interface. Although human alveologenesis begins in utero at approximately 32 weeks of gestation, a stage independent of air inflation, it is striking that we observe these alveologenesis events in the absence of forces derived from breathing movements and pressure exerted through the prenatal lung fluid. The histological changes observed in PCLS are consistent with the expansion of surface area observed in vivo (Figure 1) (18), strongly buttressing this ex vivo system as a relevant model for exploring many of the core features of alveologenesis. Additional strengths of this model include that PCLS contain the relevant parenchymal cell types of the lung, including those producing matrix and growth factors to support growth in medium (28) in a self-organizing tissue system that does not require exogenous additives that could have confounding influences on the system.

In summary, this 4D ex vivo imaging system is well positioned not only to further advance our understanding of the molecular and cellular mechanisms of alveologenesis, but also for future work evaluating effects of developmental or toxicological lung injury on alveologenesis. We envision future work leveraging this platform for therapeutically relevant drug discovery, including future application to live imaging of human PCLS (29). Our computational modeling of the timing and geometry of individual and combined alveologenesis events provides a framework for measuring perturbations of this process with preclinical genetic models, environmental exposures, and pharmacologic therapies. An immediate translational opportunity lies in testing the library of drugs commonly used in the neonatal intensive care unit to determine whether these therapeutics are associated with promotion or inhibition of alveolar growth and repair.

## Methods

### Sex as a biological variable.

The experiments reported here were performed on equal numbers of male and female mice within treatment conditions and time points, with no discernible differences noted or quantified between sexes.

### Animal sample collection.

C57BL/6J mice were used for all experiments. Timed matings were performed as described previously (5), with animals sacrificed at P5, P7, P10, and P14 as indicated.

### Fluorescently labeled PCLS.

The C57BL/6J background was used for all mouse experiments. For fluorescence imaging, Nkx2-1-Cre (stock 008661) (30), Pdgfra-Cre (stock 013148) (31), Shh-Cre (stock 005622) (32), Sftpc-CreER^T2^ (stock 028054) (14), or Ager-CreER^T2^ (stock 036942) (33) mice were crossed with mT/mG mice (stock 007676) (13) (all from The Jackson Laboratory), to generate Nkx-Cre;mT/mG, Pdgfra-Cre;mT/mG, Shh-Cre;mT/mG, Sftpc-CreER^T2^;mT/mG, or Ager-CreER^T2^;mT/mG mice. Primers used for the verification of mouse genotypes are described in Table 1, or genotyping was performed by Transnetyx using real-time PCR. For collection of lung tissues, mice were sacrificed on P5 for creation of PCLS. When needed, mice were given intraperitoneal injections of tamoxifen suspended in corn oil at 150 μg/g either on P3 and P4, or P12 and P13. PCLS were created as described previously (11). Briefly, lungs were inflated with low-melt-temperature agarose and sliced on a vibratome to a thickness of 300–400 μm. Slices were washed in DMEM/F-12 medium with penicillin/streptomycin, and then transferred to DMEM/F-12 without phenol red and with penicillin/streptomycin for imaging. When needed, nuclei were stained with Janelia Fluor 646 at a concentration of 300 μM in the imaging (34) or actin was stained with SPY650-FastAct (Cytoskeleton Inc.). Tissues were maintained at 37°C, 5% CO_2_, and atmospheric oxygen.

### Tissue clearing.

PCLS were fixed overnight in 4% methanol-free paraformaldehyde (PFA) (Electron Microscopy Sciences) in PBS prior to clearing using the Passive CLARITY method (35). Briefly, samples were washed in PBS and then incubated overnight at 4°C in A4P0 solution composed of 4% acrylamide (Bio-Rad Laboratories) and 0.25% 2,2′-azobis[2-(2-imidazolin-2-yl)propane] dihydrochloride (Wako, VA-044) in PBS. Samples were incubated at 37°C for 4 hours to polymerize the hydrogel, followed by washing with PBS. Samples were incubated in 8% SDS (pH 7.5) for 48 hours at 37°C with shaking, followed by extensive washing in PBS. PCLS were then stained by immunofluorescence or hybridization chain reaction as needed.

### Lung morphometry.

To assess development in PCLS, tissues were fixed in 10% phosphate-buffered formalin at the time of creation (+0 hours) or 48 hours later. Fixed tissues were embedded in paraffin and thin sections were stained with H&E. Alveolar septal tip length was measured as described previously (36).

### Light-sheet imaging with SOPi microscopy.

Volumetric time-lapse imaging was performed via 2 versions of the SOPi microscope platform (the second system being a modified version of the first), which were built based on the original design by Kumar et al. (4, 37). In brief, the oblique plane microscopy (OPM) class of light-sheet platforms excite specimens at an oblique angle of incidence from a single objective at the sample, serving both excitation and detection functions. By sweeping the angle of incidence of an offset, line-focused, excitation beam relative to the back focal plane of this objective, pure translation of an oblique planar (“light sheet”) excitation profile can be achieved at the sample. Thus, this approach maintains the low overall sample irradiance and high-speed capabilities inherent to many light-sheet designs, while remarkably increasing sample flexibility and mounting practicality when compared with approaches requiring 2 (or more) orthogonally oriented objectives at the sample. Additionally, due to the optical scanning (vs. stage scanning) approach, mechanical artifacts due to sample motion at high speed are mitigated. While the emission-side remote focus arrangement of OPM systems can be less efficient, with lower overall system numerical aperture (NA), the tradeoff of (a) increased sample flexibility, (b) high-speed volumetric imaging without the need to mechanically step the sample, and (c) lower overall system complexity, results in a more practical solution for many nonconventional samples (such as PCLS).

We have made several updates to increase resolving power, reduce aberrations, and improve overall system sensitivity over the first SOPi build. First, the re-imaging objectives in the second and third microscope arms (MO2 and MO3) were replaced with the same objective type used for the primary, MO1, objective (Olympus, 20×, NA 1.0 WI, XLUMPLFLN20XW). This modification results in a higher effective NA of the system, thus increasing resolving power over the original design and reducing aberrations associated with mismatch between first and second scope arms. In order to accommodate these updated (water immersion–based) re-imaging optics, a custom water chamber was fabricated for use between MO2 and MO3 objectives. Next, the sensor was also swapped for an Orca Fusion-BT sCMOS (Hamamatsu). A longer (*f* = 400 mm; ACT508-400-A-ML, ThorLabs) achromatic doublet lens was installed prior to the updated sensor to increase spatial sampling of the image, in line with Nyquist criterion for the increased NA and pixel size (6.5 μm vs. 11 μm on previous sensor). Excitation of fluorescence was accomplished by collimating the output of a single-mode fiber-based beam combiner (Galaxy, Coherent, Inc.) coupling 488-, 561-, and 640-nm OBIS CW lasers (Coherent, Inc). Optical scanning of the light sheet was enabled by a large beam diameter, single axis, galvanometer (ThorLabs, Inc.). Sample finding and multipoint positioning was made substantially easier by integrating a separate transmitted light imaging path just prior to MO2, via piezoelectric slider (ELL6K, ThorLabs) and mirror (PFR10-P01, Thorlabs) on a custom 90° mount, *f* = 100 mm (AC254-100-A-ML, ThorLabs) lens, and small format monochrome CMOS camera (CS165MU1, ThorLabs). A simple transmitted light path was supplied by a low-power, collimated green LED beneath the sample stage. Sample placement and multipoint imaging (*x*, *y*, *z*) over time was possible via an automated (*x*, *y*) scanning stage and dual (*z*) linear stages (Applied Scientific Instrumentation). Multipoint imaging proceeded via each sample position being volumetrically scanned in multiple channels over the multiday imaging runs. This was both utilized for disparate location sampling, as well as in (adjacent overlapping) positions required for image stitching applications and increase in field of view (both laterally and axially). Live samples were environmentally controlled via stage-top incubator regulating temperature (37°C), humidity (saturated, noncondensing), and 5% CO_2_ (Tokai Hit Co. LtD). Emission filtering for multichannel experimentation was accomplished via a triggerable emission filter wheel (Finger Lakes Instrumentation) and 525/50-, 593/46-, 615/20-, 679/41-, and 527/645-nm emission filters (Semrock/Idex). Hardware triggering and timing between sensor, galvanometer, lasers, and filter wheel was coordinated via multifunction I/O board (PCIe-6353, National Instruments). Hardware integration and image acquisition was managed via NIS-Elements software (Nikon Instruments, Inc.) and Z8G4 workstation (HP, Inc) configured with 2 Intel 6244 CPU’s, 196 GB RAM, solid state memory, and Quadro RTX-6000 GPU due to markedly increased computational demand incurred by size and nature of datasets. Such data represent volumetric imaging (~779 images/stack, with 0.45 μm/step) in multiple channels, at multiple stage coordinates, over several days of imaging. In total, PCLS were observed over 2,200 hours of imaging, with 3–5 biological replicates for each genotype and culture condition.

### Image acquisition and analysis.

A multithreaded Python-based image processing pipeline was used to efficiently process the large-scale SOPi imaging data. Raw data from the microscope was first de-noised in Nikon NIS-Elements, and then de-skewed with an affine transform using the SciPy python package or the CuPy when utilizing GPU acceleration (38, 39) and re-saved in the OME-NGFF file format (40). Small drifts over the imaging time course were corrected by calculating the phase cross correlation between time points using the algorithm implemented in the Scikit-Image package (41) followed by adjusting the image location. 3D fluorescence stills and videos were created with Bitplane Imaris 10.0.1 (Oxford Instruments). Surfaces and cellular segmentations were determined by local thresholding, with a neighborhood of one-third of the imaging width (*x* axis) to account for the gaussian nature of the light sheet, followed by watershed segmentation to separate nearby discreet cells. Surfaces were determined using the marching cubes algorithm implemented in Scikit-Image. Nuclei were segmentized using Cellpose (42), and individual nuclei (or cells) were tracked over time with Bayesian Tracker (btrack) (43).

### Immunofluorescence.

Immunofluorescence was performed as described previously (11). Briefly, 5-μm-thick formalin-fixed, paraffin-embedded tissue samples on slides were deparaffinized and blocked with SeaBlock (Thermo Fisher Scientific). Samples were stained with primary antibodies against GFP (1:100; ab13970, Abcam) and Ki67 (1:100; MA5-14520, Thermo Fisher Scientific) followed by nuclear staining with DAPI. Images were acquired on a Keyence BZX-800 or a Nikon TiE inverted spinning disk confocal microscope outfitted with a Yokogawa X1 spinning disk head and a Photometrics Prime 95B camera. Quantification was performed using HALO software (Indica Labs) on images where the Ki67 signal was segmented to be counted only in nuclear areas.

### Computational model.

Quantitative analysis of the alveologenesis data was performed on coordinates (*t*, *x*, *y*, *z*) of the inner boundaries of the alveolar bed. This data was separated by distinct time points resulting in *k* partitions of the data, with each partition representing a mathematical snapshot of the shape of the alveolus boundaries at a particular time. Each snapshot (*t* = *T*) was analyzed by choosing a particular fixed value (*z* = *Z*) and studying the set of planar points (*T*, *x*, *y*, *Z*). Starting with the *t* = 0 snapshot, closed, nonintersecting sets of points were identified. The set with the largest interior area was chosen as the alveolus of interest. At each instant of time, the set of points was approximated by a polygon at *z* = *Z*. The area of its interior and the perimeter length were calculated for the polygon. A program was written in Mathematica (Wolfram) to perform these calculations, including fitting the set of points with a polygon. The sets {(*t_i_*, *A_i_*), *I* = 1, 2…, *k*} and {(*t_i_*, *P_i_*), *I* = 1, 2, ….*k*} were generated, which describe the evolution of area and perimeter length over time. Another measure of growth/decay is the area divided by the perimeter length, and the area-to-perimeter ratio is an indicator of polygon shape complexity and is the opposite of compactness.

### SEM.

Samples were fixed in 2% PFA and 2% glutaraldehyde followed by sequential postfixation in 1% tannic acid, 1% OsO_4_, and 1% uranyl acetate. The samples were then dehydrated in a graded ethanol series and critical point dried. Samples were fractured using a scalpel and coated with 2-nm Pt and 3-nm carbon using a Leica ACE600 ebeam system. SEM was performed using a Zeiss Crossbeam 550 FIB-SEM.

### Data availability.

All numerical data used in graphs are available in the Supporting Data Values file uploaded as a supplement. Raw data from the imaging movies are available upon request.

### Study approval.

All animal work was approved by the Institutional Animal Care and Use Committee of Vanderbilt University (Nashville, Tennessee, USA) and was in compliance with the NIH *Guide for the Care and Use of Laboratory Animals* (National Academies Press, 2011).

### Statistics.

All statistical analysis was performed with GraphPad Prism (version 10.3.1, 2024). When comparing 2 groups, Student’s *t* test was used, and when comparing multiple groups, 1-way analysis of variance (ANOVA) was used with Bonferroni’s correction for multiple comparisons.

### Data and materials availability.

All imaging data is available upon request. The code used for transforming and processing the SOPi imaging data is available at https://github.com/SucreLab/SOPi_Alveologenesis

## Author contributions

NMN, BM, and JMSS conceptualized the study and curated data. NMN, YS, and JMSS analyzed data. JMSS and BM acquired funding. NMN, PC, YS, ANH, MR, DN, CSJ, CB, and JMSS conducted experiments. SG conducted experiments, edited the manuscript, and analyzed data. EJP, JTB, JMSS, BM, NMN, NM, AMJ, JA Kozub, and EK developed methodology. HP developed methodology and edited the manuscript. CSJ provided project administration. NMN, BM, and PC provided software. NMN, EJP, JTB, JA Kropski, TSB, SHG, JMSS, ANH, and MR validated results. NMN, BM, and YS generated figures. NMN and JMSS wrote the original draft of the manuscript, which was reviewed and edited by NMN, BM, YS, PC, EJP, DBF, WZ, CVEW, JTB, SHG, TSB, JA Kropski, and JMSS. JMSS and BM supervised the study.

## Supplementary Material

Supplemental data

Supplemental video 1

Supplemental video 2

Supplemental video 3

Supplemental video 4

Supplemental video 5

Supplemental video 6

Supporting data values

## Figures and Tables

**Table 1:**
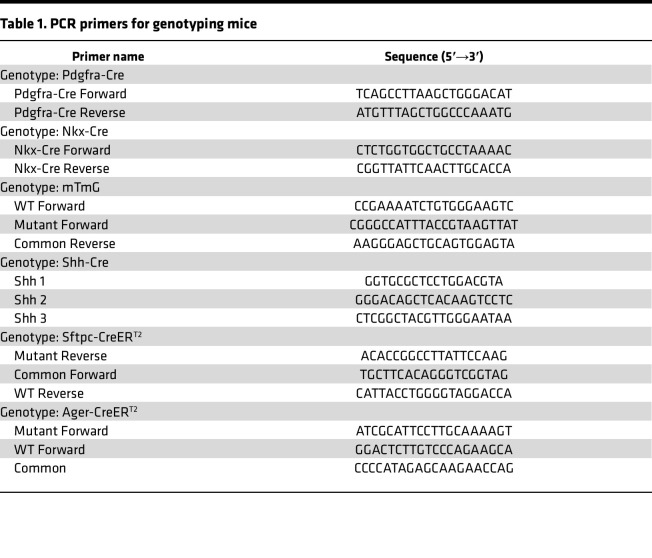
PCR primers for genotyping mice

## Version 1 01/07/2025

In-Press Preview

## Version 2 02/24/2025

Electronic publication
