# Supplementary material for: Epithelial outgrowth through mesenchymal rings drives lung alveologenesis
Source: JCI Insight. 2025 Jan 7;10(4):e187876. doi: 10.1172/jci.insight.187876 (PMC11949025; doi:10.1172/jci.insight.187876)
Supplement: Supplemental data [file jciinsight-10-187876-s230.pdf]

SUPPLEMENTARY FIGURE LEGENDS

Figure S1. Agarose is present in the large airways of PCLS, but not in the distal alveolar airspaces. A) Fluorescein-labeled agarose in PCLS imaged by confocal microscopy using fluorescence and phase contrast imaging. Scale bar = 100 µm. **B)** Scanning electron microscopy of the surface of a PCLS. Scale bar = 20 µm. **C)** Airspace volume density was calculated and compared from PCLS created from the same lung at P5 and P5 + 48 hours (six mice from two litters), with structural changes equivalent in many respects to **D)** the sections from P5 or P7 mice. ***p<0.001*,* ****p*<0.*0001.

Figure S2. The incidence of Sftpc-traced cell-shape change decreases in PCLS during later alveologenesis. A) Still frames from 4D imaging of PCLS from mT/mG;Sftpc-CreERT2 mice given tamoxifen on P3 and P4 and imaged at P7. **()** Still frames from 4D imaging of PCLS from mT/mG;Sftpc-CreERT2 mice given tamoxifen on P3 and P4 and imaged at P14. **C)** Still frames from 4D imaging of PCLS from mT/mG;Sftpc-CreERT2 mice given tamoxifen on P12 and P13 and imaged at P14. **D)** Quantification of alveologenesis events per hour in PCLS made at P5 and P14.

Figure S3. Similar patterns of epithelial cell movement and shape change are consistent across Cre drivers. A) Still frames from 4D imaging of PCLS from mT/mG;Shh-Cre and **B)** mT/mG;Nkx2.1-Cre mice. Arrows indicate cells undergoing movement and shape change. **C)** Immunostaining for Ki67 in P5 and P7 mice indicates that few *Sftpc*+ cells co-stain with Ki67 at P5 or P7. **D**) Quantification of percentage of Ki67+*Sftpc*+ double-positive cells out of all *Sftpc*+ cells. **E)** Single-cell sequencing data from AT2 cells over the course of mouse lung development represented by dot plot also indicates a low proportion of *Mki67*+ AT2 cells after birth in mice.

Figure S4. Pdgfra+ labeled cells gradually lose their ring shape over the course of the alveolar stage, with live imaging demonstrating decreased cellular movement with increased age at the time of imaging. A) Imaris-based surface rendering of thick, tissue-cleared sections from the lungs of mT/mG;Pdgfra-Cre mice at P7 and P10 with GFP+ cells

segmented, and identified rings colored red. Airspaces are transparent outlines. PCLS from mT/mG;Pdgfra-Cre mice were created at **B)** P10 and **C)** P14 and live imaged. **D)** Immunostaining for GFP (green), *Sftpc* (magenta), PDPN (white), and DAPI (blue) in PCLS from mT/mG;Sftpc-CreERT2 mice treated with ML-7 for 48 hrs.

Figure S5. Addition of Wnt activator and inhibitor is validated by the presence or absence of nuclear β-catenin, with quantification of cellular movements in response to manipulating the Wnt pathway. A) PCLS created at P5 from mT/mG;Sftpc-CreERT2 mice treated with CHIR or XAV were immunostained for PDPN (white), GFP (green), or β-Catenin-y489 (magenta), with DAPI counterstaining (blue). **B)** Processivity (a measure of directed cell motion) and speed was calculated by from the nuclear motion in live-imaged PCLS (****p<0.01 by Student's *t*-test).

SUPPLEMENTARY VIDEO LEGENDS:

SV1: PLCS from P5 mT/mG Sftpc CreERT2 mouse (tamoxifen given on P3 and P4). Over the 77 hr imaging period, there are numerous instances of rounded GFP+ cells spreading and flattening asynchronously (highlighted by white arrows). Insets: one cell shown migrating and spreading from three different angles to characterize these cellular movements.

SV2: PLCS from P5 mT/mG Sftpc CreERT2 mouse (tamoxifen given on P3 and P4). Three examples of the cellular aggregation and outgrowth occurring in these Sftpc+ cells across the lung slice.

SV3: PCLS from P5 mT/mG Pdgfra Cre mouse. 1) 39 hours of imaging with the full volumetric z-stack on the left and a single optical plane from the z-stack on the right. Comparing the single plane with the z-stack at the same timepoint, once can see that the cellular "septa" observed by 2D imaging are actually part of more complex ring structures 2) Visualization of the Pdgfra cells in 3D reveals multiple complex movements and ring formation, with some rings gradually decreasing in size over time.

SV4: PCLS from P5, 7, 10, and 14 (indicated in lower left corner) from a mT/mG Pdgfra Cre mouse that were fixed immediately and immunostained for GFP. In the initial frames, all of the Pdgfra cells are blue, with individual ring structures demonstrated in red, followed by a representation of airspaces in the setting of the rings.

SV5: PLCS from P5 mT/mG Sftpc CreERT2 mouse (tamoxifen given on P3 and P4). One example of a rounded GFP+ cell extruding through the ring structure and joining with other aggregating and spreading epithelial cells to form a neoalveolus.

SV6: PLCS from P5 mT/mG Sftpc CreERT2 mouse (tamoxifen given on P3 and P4) treated with either CHIR or XAV and imaged for 42 hrs, with only the GFP channel shown. Treatment is indicated by the text embedded in the video. We see markedly decreased movement of GFP+ cells, when compared with control conditions in SV1 and SV2.

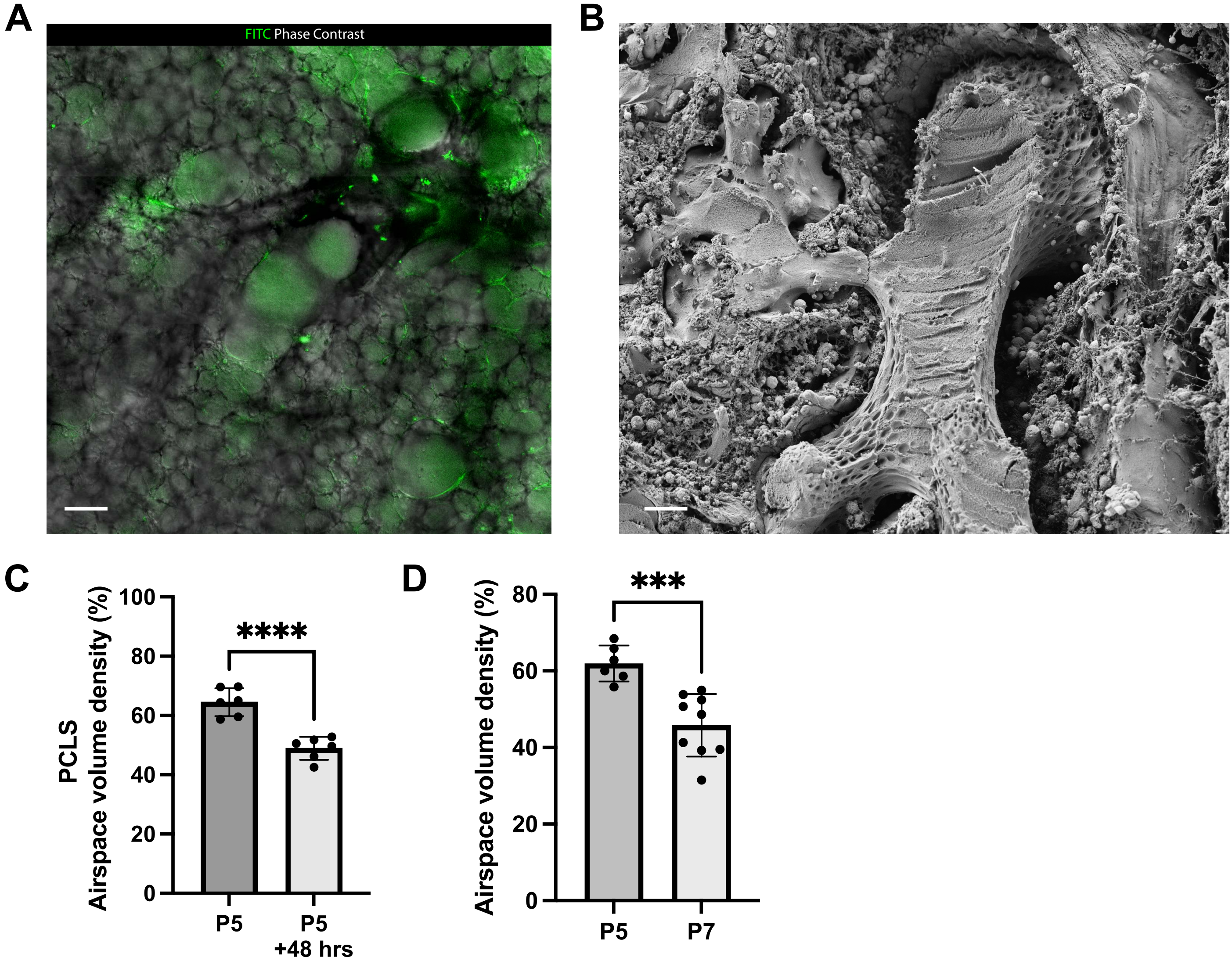
A
FITC Phase Contrast
B
C
PCLS
Airspace volume density (%)
100
80
60
40
20
0
****
P5
P5
+48 hrs
D
Airspace volume density (%)
80
60
40
20
0
***
P5
P7

Figure S1

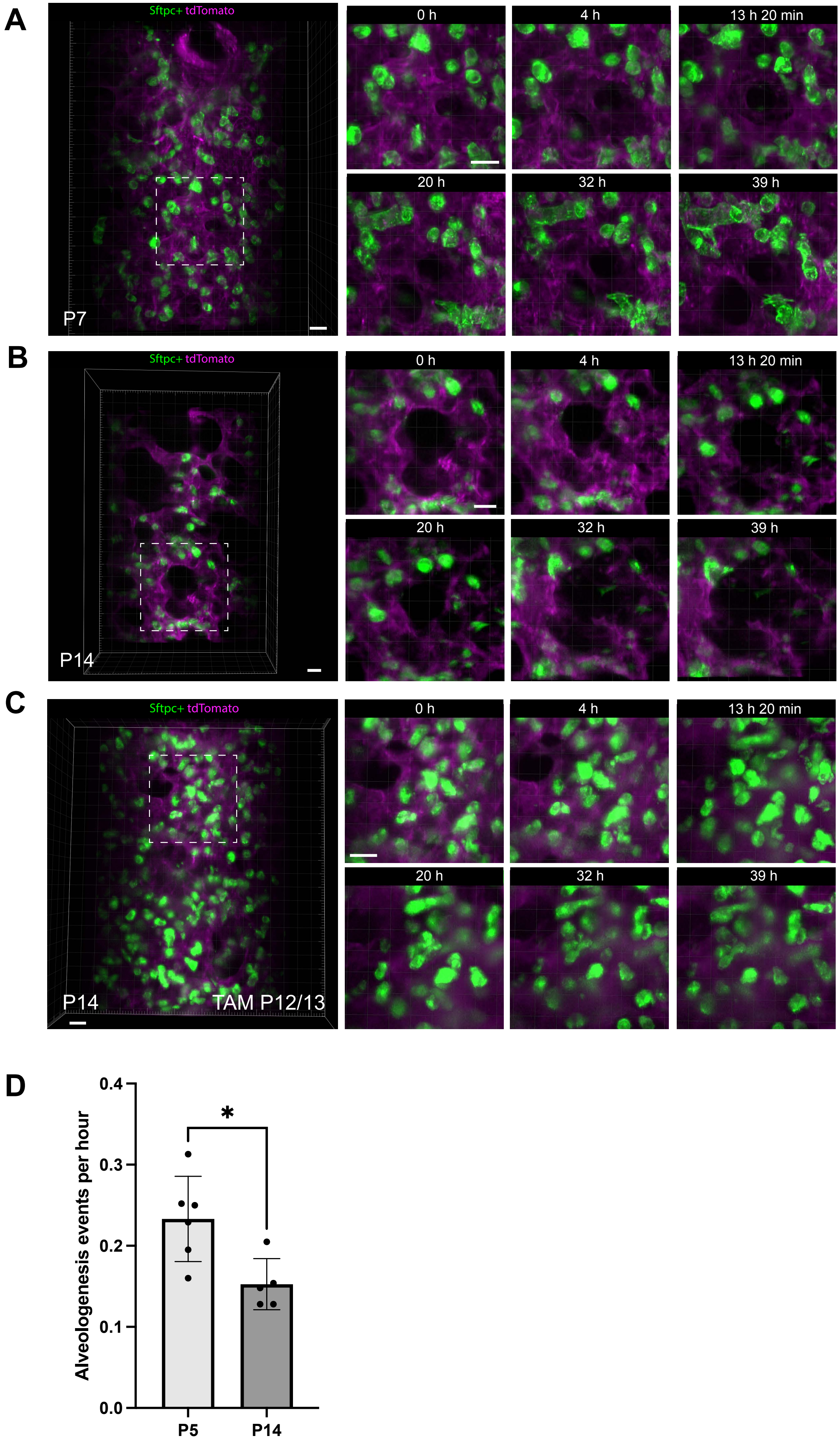
A
Sftpc+ tdTomato
P7
0 h
4 h
13 h 20 min
20 h
32 h
39 h
B
Sftpc+ tdTomato
P14
0 h
4 h
13 h 20 min
20 h
32 h
39 h
C
Sftpc+ tdTomato
P14
TAM P12/13
0 h
4 h
13 h 20 min
20 h
32 h
39 h
D
Alveologenesis events per hour
0.4
0.3
0.2
0.1
0.0
*
P5
P14

Figure S2

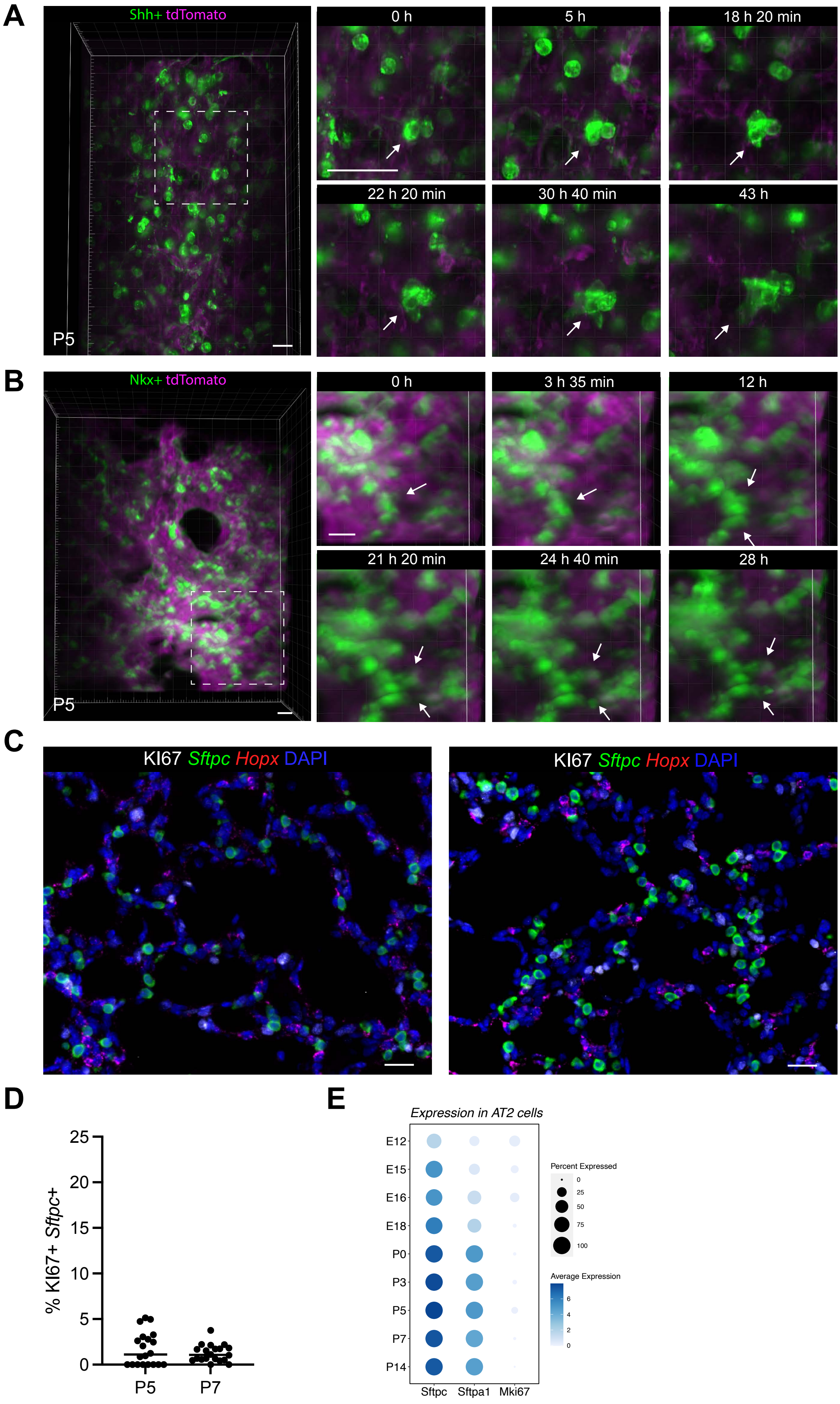
A
Shh+ tdTomato
0 h
5 h
18 h 20 min
22 h 20 min
30 h 40 min
43 h
P5
B
Nkx+ tdTomato
0 h
3 h 35 min
12 h
21 h 20 min
24 h 40 min
28 h
P5
C
KI67 Sftpc Hopx DAPI
KI67 Sftpc Hopx DAPI
D
% KI67+ Sftpc+
25
20
15
10
5
0
P5
P7
E
Expression in AT2 cells
E12
E15
E16
E18
P0
P3
P5
P7
P14
Sftpc
Sftpa1
Mki67
Percent Expressed
0
25
50
75
100
Average Expression
6
4
2
0

Figure S3

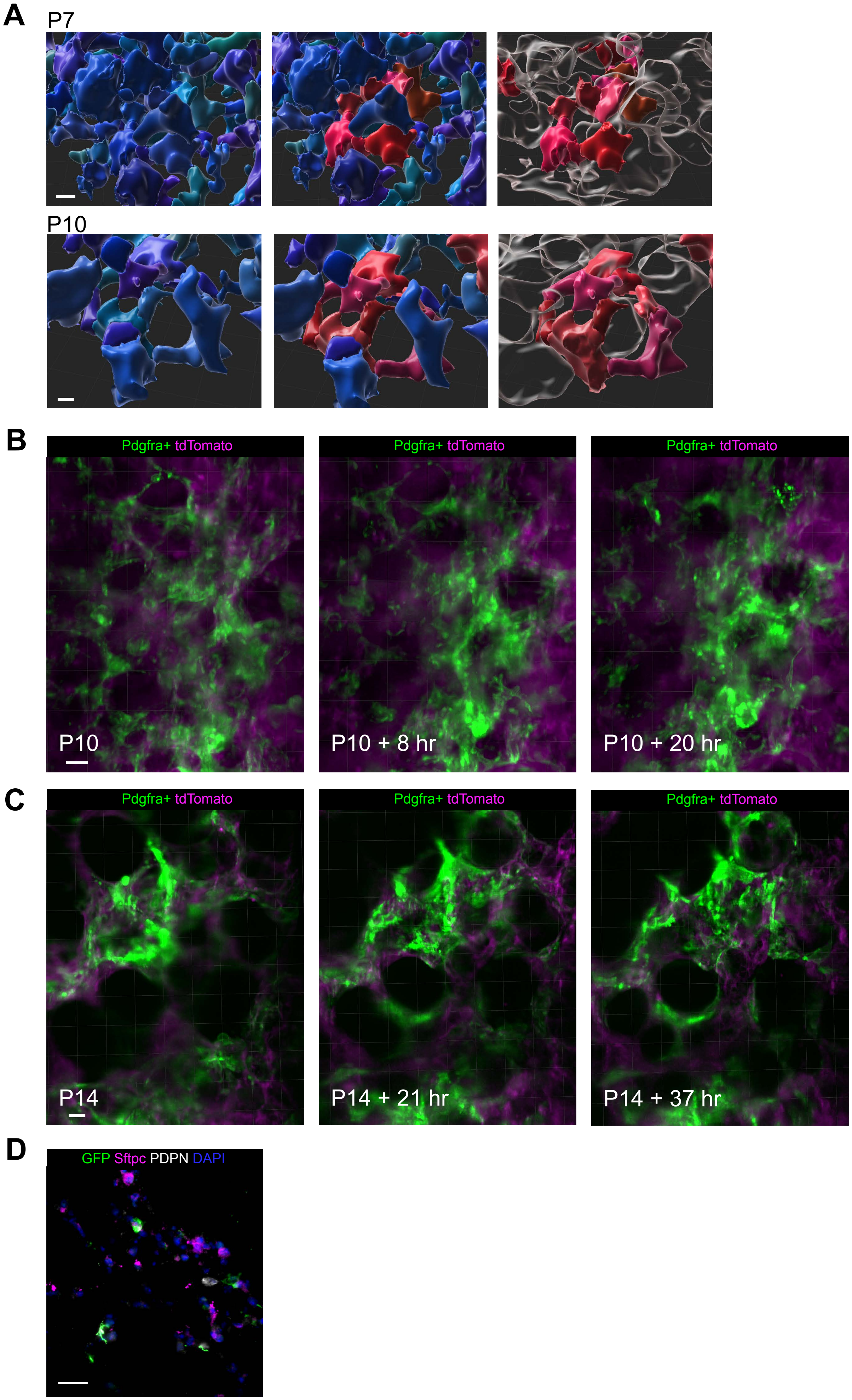
A
P7
P10
B
Pdgfra+ tdTomato
Pdgfra+ tdTomato
Pdgfra+ tdTomato
P10
P10 + 8 hr
P10 + 20 hr
C
Pdgfra+ tdTomato
Pdgfra+ tdTomato
Pdgfra+ tdTomato
P14
P14 + 21 hr
P14 + 37 hr
D
GFP Sftpc PDPN DAPI

Figure S4

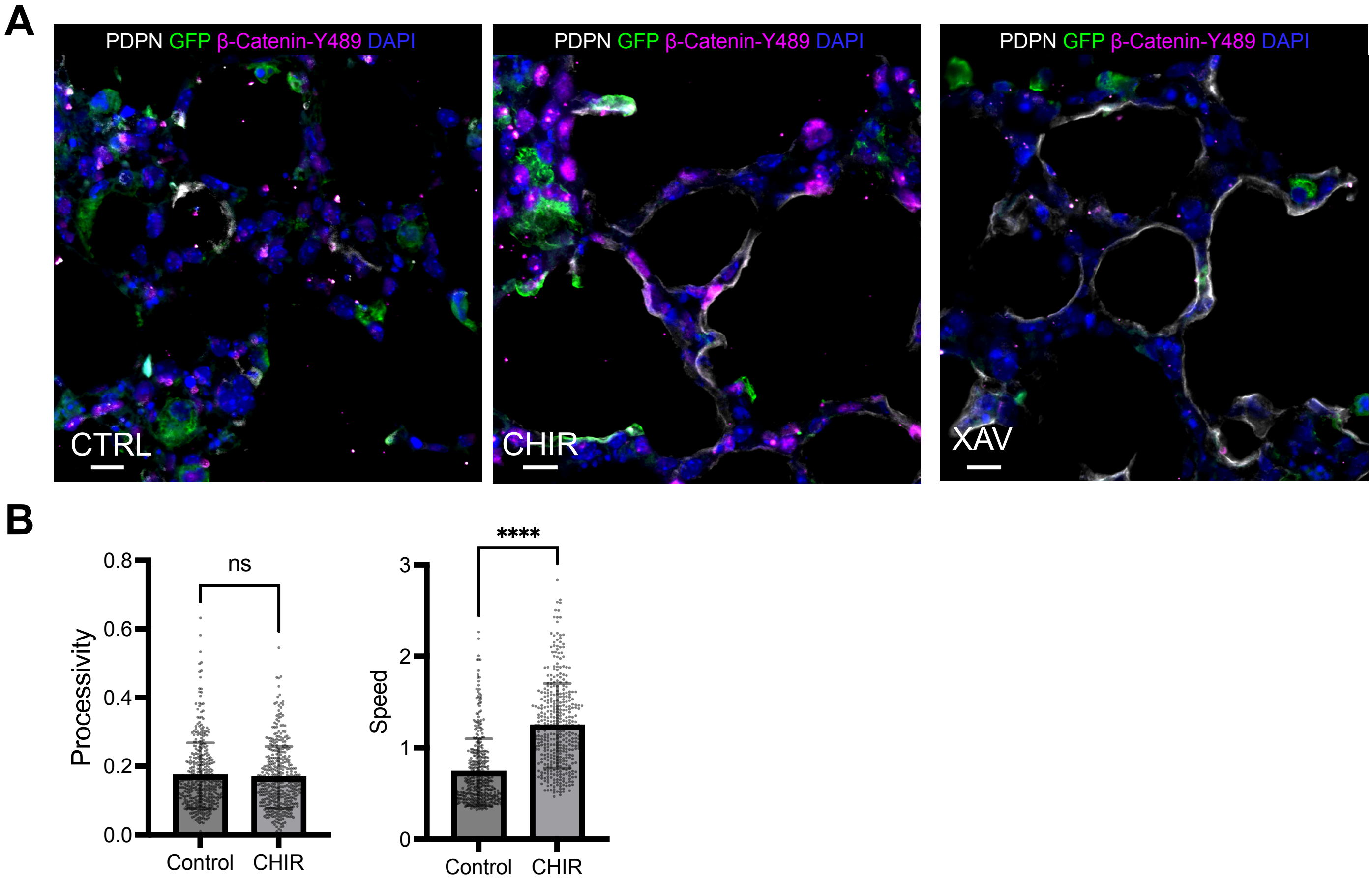
A
PDPN GFP β-Catenin-Y489 DAPI
CTRL
CHIR
XAV
B
Processivity
0.8
0.6
0.4
0.2
0.0
ns
Control
CHIR
Speed
3
2
1
0
****

Figure S5
